# Transcriptomic reprogramming and displacement of mastitis-associated *Staphylococcus aureus* biofilms by lactic acid bacteria

**DOI:** 10.3389/fcimb.2026.1957508

**Published:** 2026-10-26

**Authors:** Rubén Jurado, Angela França, Natalia Hernando, Alberto Aragón, Juan M. Rodríguez, Leonides Fernández, Belén Orgaz

**Affiliations:** 1 Dept. Galenic Pharmacy and Food Technology, Complutense University of Madrid, Madrid, Spain; 2 Centre of Biological Engineering (CEB), Laboratory of Research in Biofilms Rosário Oliveira (LIBRO), University of Minho, Braga, Portugal; 3 Multidisciplinary Institute, Complutense University of Madrid, Madrid, Spain; 4 Dept. Nutrition and Food Science, Complutense University of Madrid, Madrid, Spain

**Keywords:** biofilms, *Ligilactobacillus salivarius*, *Limosilactobacillus fermentum*, *Limosilactobacillus reuteri*, mastitis, RT-qPCR, RNA-seq, *Staphylococcus aureus*

## Abstract

*Staphylococcus aureus* is one of the main etiological agents of acute mastitis during lactation, and its ability to persist in biofilms can compromise conventional antimicrobial treatments. Lactic acid bacteria (LAB) have emerged as promising probiotic candidates for mastitis management, but the molecular mechanisms underlying their interaction with *S. aureus* remain poorly understood. This study aimed to evaluate the ability of three LAB strains (*Limosilactobacillus fermentum* I7, *Limosilactobacillus reuteri* 7SNG3–30 and *Ligilactobacillus salivarius* 22SNG3-30) to interfere with mature biofilms formed by two mastitis-associated *S. aureus* strains, SA4 and SA55, and to characterize the associated transcriptional responses. The LAB strains were able to colonize preformed 24-h *S. aureus* biofilms, eventually dominating the mixed-species communities and reducing the attached staphylococcal population. RT-qPCR showed limited transcriptional changes after 4 h of LAB exposure, followed by a broader 16-h response characterized by induction of the stress/adaptation marker *uspA* and repression of genes related to quorum sensing, virulence, biofilm matrix production and iron acquisition. RNA-seq analysis of SA4 biofilms exposed to LAB for 16 h confirmed extensive transcriptional remodeling in *S. aureus* and revealed a conserved response across the three LAB treatments, including activation of stress, proteostasis, energy metabolism and arginine catabolism functions, together with repression of surface-associated, matrix-related, metal acquisition and virulence-associated pathways. In contrast, LAB transcriptomic responses were more moderate and strain-dependent, suggesting different metabolic, nutrient-acquisition and envelope-adaptation strategies during biofilm-associated interactions. Overall, these findings suggest that these LAB strains can displace mastitis-associated *S. aureus* from mature biofilms and promote a coordinated transcriptional shift toward stress adaptation and attenuation of virulence- and persistence-associated functions.

## 1 Introduction

Lactational mastitis is a frequent and painful condition that can seriously compromise breastfeeding, and *Staphylococcus aureus* remains one of its main etiological agents (Delgado et al., 2011; Hughes and Watson, 2018). Clinical management still relies largely on empiric antibiotic therapy; however, treatment failure and recurrence are often favored by the remarkable capacity of *S. aureus* to persist in biofilms, i.e., complex multicellular structures that protect the pathogen from host immune responses and significantly reduce antimicrobial penetration (Marín et al., 2017; Angelopoulou et al., 2018; Guo et al., 2022). Within these structures, metabolic heterogeneity and the presence of stress-tolerant subpopulations, such as persister cells or small colony variants, can markedly reduce antibiotic efficacy even when planktonic cells appear susceptible (Zaatout et al., 2020; Guo et al., 2022). Furthermore, antibiotic use during lactation raises additional concerns, including possible disruption of the infant’s developing microbiota and the broader selective pressure that contributes to antimicrobial resistance (Cox et al., 2014; Fernández et al., 2016; Jiménez et al., 2021; Lynch et al., 2023). Together, these limitations highlight the need for alternative or complementary approaches capable of controlling biofilm-embedded *S. aureus* while remaining compatible with breastfeeding.

In this context, lactic acid bacteria (LAB), particularly strains originating from human milk environments, have emerged as promising probiotic candidates for the prevention or management of infectious mastitis. Randomized clinical trials have shown that certain lactobacilli strains can reduce staphylococcal loads and improve clinical symptoms, in some cases more effectively than antibiotic therapy (Jiménez et al., 2008; Arroyo et al., 2010; Fernández et al., 2016; Jiménez et al., 2021). In parallel, experimental studies have demonstrated that LAB and LAB-derived products can inhibit *S. aureus* growth and biofilm formation (Kang et al., 2017; Mao et al., 2023; Jurado et al., 2025b). These findings support the idea that LAB may interfere with pathogen persistence through multiple mechanisms mediated by secreted antimicrobial and antibiofilm metabolites (Jiménez et al., 2008; Kang et al., 2017; Mao et al., 2023). Nonetheless, the available evidence has remained largely phenotypic, focusing mainly on viable counts, overall antibiofilm activity, or clinical outcomes rather than on the transcriptional responses of the pathogen. Consequently, global transcriptional and molecular pathways triggered by mastitis-associated *S. aureus* strains during direct interaction with LAB strains remain a critical knowledge gap (Viçosa et al., 2018; Jiménez et al., 2021).

Understanding how LAB affect *S. aureus* in the biofilm state is particularly relevant because biofilm development is governed by regulatory networks that integrate quorum sensing, metabolic adaptation, stress responses and virulence control (Bronesky et al., 2016; Jenul and Horswill, 2019; Guo et al., 2022). Among these, the *agr* (accessory gene regulator) system and its downstream RNAIII regulon play central roles in coordinating the transition between surface attachment, toxin production, biofilm maturation and dispersal, while additional pathways involved in autolysis, iron acquisition, proteolysis, secretion and oxidative stress contribute to persistence in hostile niches (Boles and Horswill, 2011; Wang and Muir, 2016; Guo et al., 2022; Mujchariyakul et al., 2026). The few available transcriptomic studies have generally been conducted in non-biofilm experimental systems using planktonic cocultures with *Lactococcus lactis* and food-based models (Even et al., 2009; Cretenet et al., 2011; Viçosa et al., 2018; Peng et al., 2022). Moreover, mechanistic work in mastitis-related systems has focused on host-side outcomes, particularly in ruminant-based models (Cremonesi et al., 2012; Bouchard et al., 2013; Gilbert et al., 2013; Fukuyama et al., 2020; Saleem et al., 2024). Thus, the transcriptional basis of the interactions between LAB and mastitis-associated *S. aureus* strains in biofilms remains poorly understood.

Here, we combined phenotypic and transcriptomic approaches to investigate whether potentially probiotic LAB could interfere with the persistence of established *S. aureus* biofilms. We evaluated three LAB strains recovered from human milk and neonatal milk-feeding environments: *Limosilactobacillus fermentum* I7, isolated directly from human milk, and *Limosilactobacillus reuteri* 7SNG3–30 and *Ligilactobacillus salivarius* 22SNG3-30, isolated from biofilms colonizing the inner surfaces of nasogastric enteral feeding tubes used to deliver milk to preterm infants (Jara Pérez et al., 2022). The tube-derived isolates were selected because their recovery from these milk-exposed surface-associated communities indicated an ability to colonize and persist within biofilms, a potentially advantageous trait for competing with mature *S. aureus* biofilms. The three LAB strains were evaluated against 24-h biofilms formed by two acute mastitis-associated *S. aureus* strains, SA4 and SA55. After confirming antibiofilm activity, RT-qPCR analysis revealed consistent transcriptional changes in *S. aureus* pathways linked to adhesion and matrix dynamics, quorum sensing and virulence regulation, stress adaptation, and secreted effectors after early and prolonged LAB exposure. To further address and characterize the transcriptomic responses of *S. aureus* SA4 and the three LAB strains during biofilm-associated interspecies interaction, global transcriptomic profiling was performed by RNA sequencing (RNA-seq).

## 2 Materials and methods

### 2.1 Bacterial strains and growth conditions

Two *S. aureus* and three LAB strains were used in this study. *S. aureus* SA4 and SA55 were isolated from the milk of women suffering from acute mastitis. The LAB strains were isolated either from human milk (*L. fermentum* I7) or from the inner surface of nasogastric enteral feeding tubes used to feed preterm infants (*L. reuteri* 7SNG3–30 and *L. salivarius* 22SNG3-30). The samples from which the strains were originally isolated were collected in the frame of previous studies (Delgado et al., 2011; Jara Pérez et al., 2022). No new participants were recruited, and no new human samples were collected for the present study, which used exclusively bacterial strains previously isolated in the aforementioned studies. The original collection of human milk samples and the corresponding experimental procedures were authorized by the Ethics Committee of Hospital Clínico San Carlos of Madrid, Spain (Code: 06/41; date: 03/16/2006), and written informed consent was obtained from all participating women. The original study involving nasogastric enteral feeding tubes from preterm infants received approval from the Ethics Committee on Clinical Research of La Paz University Hospital of Madrid, Spain (Code: HULP PI-3199; date: 06/27/2018), and written consent was provided by the legal guardians before sample and data collection. Both studies were conducted in compliance with the principles of the Declaration of Helsinki. Unless otherwise indicated, *S. aureus* strains were cultured in Brain Heart Infusion (BHI) broth (Oxoid, Basingstoke, UK), whereas LAB strains were grown in de Man, Rogosa and Sharpe (MRS) broth (Oxoid). All the cultures were incubated aerobically at 37 °C for 24 h under static conditions.

### 2.2 Treatment of *S. aureus* biofilms with active cultures of LAB strains

Preformed *S. aureus* biofilms were generated in sterile 24-well Nunclon™ Delta surface polystyrene plates (Thermo Scientific™, Roskilde, Denmark). Briefly, an isolated colony of each *S. aureus* strain was inoculated into 10 mL of BHI broth and incubated overnight at 37 °C under static conditions. Overnight cultures were harvested by centrifugation (5,000 × *g*, 15 min, 4 °C), washed once with fresh BHI, and resuspended in BHI to achieve an optical density at 600 nm (OD_600_) of ~0.1, corresponding to approximately 10^7^ CFU (colony-forming units)/mL. For biofilm formation, 990 µL of BHI broth were dispensed into each well and inoculated with 10 µL of the standardized *S. aureus* suspension, resulting in an initial concentration of approximately 10^5^ CFU/mL per well, and plates were incubated aerobically at 37 °C for 24 h.

After biofilm development, the supernatant was carefully removed, and each well was gently rinsed once with sterile saline solution (0.85% NaCl, w/v) to eliminate non-adherent cells. Then, 1 mL of either MRS broth (negative control) or 1 mL of MRS broth supplemented with active LAB cultures at a final concentration of approximately 10^5^ CFU/mL (either *L. fermentum* I7, *L. reuteri* 7SNG3-30, or *L. salivarius* 22SNG3-30) was added to the preformed *S. aureus* biofilms. LAB inocula were prepared following the same standardization procedure described for *S. aureus*. Each of the four conditions (MRS control and one condition for LAB strain) was assessed by triplicate. Plates were incubated aerobically at 37 °C for an additional 24 h.

To assess the number of culturable cells in pre-established *S. aureus* biofilms, supernatants were discarded, wells were rinsed once with sterile saline solution, and adherent cells were recovered by thoroughly scraping the well bottom and inner surface with a sterile swab. Swabs were transferred to 1 mL of sterile peptone water, vigorously vortexed to detach cells, and the resulting suspensions were serially diluted. Aliquots (20 µL) of appropriate dilutions were plated onto selective media: MRS agar for LAB counts and Baird-Parker (BP; Pronadisa, Spain) for staphylococcal counts. Plates were incubated at 37 °C for 24 h, and results were expressed as log_10_ CFU/cm^2^. The theoretical limit of detection was 1.42 log_10_ CFU/cm^2^. The number of culturable cells in the 24-h preformed *S. aureus* biofilms was quantified immediately before LAB exposure and every 4 h post-LAB exposure for up to 24 h.

### 2.3 RNA extraction and quantitative real-time PCR

*S. aureus* biofilms were processed immediately after 4 h or 16 h of exposure to the corresponding LAB treatment. After removing the culture medium, wells were gently washed once with sterile saline solution, immediately stabilized by adding 250 µL of RNAprotect® Bacteria Reagent per well (QIAGEN, Hilden, Germany) (previously diluted 2:1 in water), and biofilm-associated cells were recovered by scraping the well and inner surface. For each independent biological replicate, biofilms from four technical replicate wells per condition were pooled to obtain sufficient RNA for downstream analyses. Total RNA was extracted using the RNeasy® Mini Kit (QIAGEN). Before RNA isolation, cells were mechanically disrupted with 150–212 µm acid-washed glass beads (Merck, Darmstadt, Germany) in a bead-beating homogenizer (BeadBug 6 Microtube Homogenizer, Benchmark Scientific, NJ, USA) for three cycles of 30 seconds at 4,350 rpm. Clarified lysates were processed according to the manufacturer’s protocol, including on-column DNase digestion (RNase-Free DNase Set, QIAGEN), and RNA was eluted in nuclease-free water. RNA concentration and purity were assessed with a NanoDrop spectrophotometer, and RNA integrity was evaluated by native agarose gel electrophoresis.

Complementary DNA (cDNA) was synthesized from 300 ng of total RNA using the iScript™ gDNA Clear cDNA Synthesis Kit (Bio-Rad Laboratories, Hercules, CA, USA) according to the manufacturer’s instructions. Reverse transcription negative controls (no-RT) were included to determine the level of genomic DNA contamination. Quantitative PCR (qPCR) was carried out using SsoAdvanced™ Universal Inhibitor-Tolerant SYBR® Green Supermix (Bio-Rad Laboratories) on a CFX Opus 96 real-time PCR system (Bio-Rad Laboratories). Each reaction (10 µL) contained 5 µL of SsoAdvanced Supermix, 0.5 µL of forward primer, 0.5 µL of reverse primer, 2 µL of nuclease-free water, and 2 µL of cDNA template diluted 1:100. No-template controls (NTC) were included in each run to assess reagent contamination. The thermal cycling program was as follows: initial denaturation/activation at 98 °C for 3 min, followed by 39 cycles of 98 °C for 15 s and 60 °C for 35 s, and a melt-curve analysis from 65 to 95 °C with 1 °C increments. All qPCR reactions were run in technical triplicate, and the resulting Ct values were averaged for each gene and biological replicate before relative gene-expression analysis.

Primer pairs targeted 13 *S. aureus* genes associated with biofilm formation, stress response, virulence regulation, secretion/proteolysis and quorum sensing, while *16S rRNA* and *gyrB* were used as reference genes. Primers were designed using Primer3 (v4.1.0) (Untergasser et al., 2012). Primer sequences, amplicon sizes and amplification efficiencies are summarized in **Table 1**. Primer specificity and/or primer-dimer formation was evaluated by melt-curve analysis. Relative gene expression was calculated using the 2−ΔΔCt method after normalization to the geometric mean of the two reference genes (*16S rRNA* and *gyrB*), whose expression stability across the tested conditions was evaluated using RefFinder (Xie et al., 2012).

**Table 1:** List of primers used in this study.

Target gene	Primer sequence (5′-3′)	Amplicon size (bp)	Efficiency (%)	Function	Reference
*16S rRNA*	F: CCGCATGGTTCAAAAGTGA	100	100.6	Reference gene	This study
	R: ATCGTTGCCTTGGTAAGCC				
*gyrB*	F: TCCTATCACTATGAGGGCGG	212	92.5	Reference gene	This study
	R: AATCCGTCTTCATGCGTACC				
*agrB*	F: GTGAGGAGAGTGGTGTAAAATTGA	119	104.1	Quorum sensing (*agr* system)	This study
	R: CATCCCTAATCGTACTTGCAAAA				
*sarA*	F: TGTTTGCTTCAGTGATTCGTTT	233	98.3	Global regulator (virulence/biofilm)	(Magalhães et al., 2019)
	R: CATCAGCGAAAACAAAGAGAAA				
*hld*	F: TTTTTAGTGAATTTGTTCACTGTGTC	100	93.1	Delta-hemolysin	(Magalhães et al., 2019)
	R: TAATTAAGGAAGGAGTGATTTCAATG				
*psmβ1*	F: ACGCAATTAAAGATACCGTAACTG	92	106.9	Phenol soluble modulin beta 1	This study
	R: ACCTACGCCATTTTCAACGA				
*seg*	F: GGAGGTTGTTGTATGTATGGTGG	181	104.4	Extracellular enterotoxin type G	This study
	R: CTTTAGTGAGCCAGTGTCTTGC				
*sspA*	F: GTGAGTTCTCCAGCAGCAAA	183	96.1	V8 protease	This study
	R: TGTATCTGTGATTTGGTGACGA				
*esaA*	F: GACGCTAAAACACCATCGAAAA	215	96.9	Secretion system (type VII)	This study
	R: CTATTCACACCATGTTCACGC				
*icaA*	F: AAGTATGAACCGCTTGCCAT	139	100.0	Biofilm formation	This study
	R: ACCTTTTCGTTTTCATTGTGCT				
*atl*	F: CGCTTGTAGGTTCAGCAGTC	194	97.4	Autolysin/biofilm remodeling	This study
	R: TGGTTGGACAATAGCAGGGT				
*sfaA*	F: TTGGATTGGACAAATAGGGCT	121	100.2	Iron acquisition (staphyloferrin A export)	This study
	R: CCCTGCCCACACACTTAGTA				
*sodA*	F: ATGGCGGTGGTCATTTTAAC	171	103.5	Stress response (superoxide dismutase)	(Magalhães et al., 2019)
	R: ACCAAGTCCAACCTGATCCA				
*uspA*	F: ACTTACTGAAGAACGAAGCAAGC	232	95.5	Stress response	(Magalhães et al., 2021)
	R: AGGGATAGTCGCACGTTTTG				
*clpP*	F: AACAACAAATCGCGGTGAAC	265	106.2	Protease (proteostasis/stress response)	(Magalhães et al., 2021)
	R: TGCAGCCATACCGATACAAA				

For each *S. aureus* strain and sampling time, LAB-treated biofilms were compared with the corresponding MRS-treated control from the same independent experiment. Each condition included four or five independent biological replicates. The resulting fold-change values were log_2_-transformed, yielding one log_2_ fold-change (log2FC) value for each biological replicate, which was used as an independent observation in the statistical analysis.

### 2.4 RNA-seq library preparation, sequencing and bioinformatic analysis

To capture global transcriptional responses during LAB–*S. aureus* interactions, RNA-seq was performed on biofilm samples collected after 16 h exposure to the LAB active cultures. The experimental design comprised seven conditions, each analyzed in three independent biological replicates: (i) SA4 control biofilms exposed to MRS alone, single-species biofilms formed by (ii) *L. fermentum* I7, (iii) *L. reuteri* 7SNG3–30 and (iv) *L. salivarius* 22SNG3-30, and preformed SA4 biofilms exposed to (v) *L. fermentum* I7, (vi) *L. reuteri* 7SNG3–30 and (vii) *L. salivarius* 22SNG3–30 for 16 h. The three biological replicates were generated from independently initiated biofilm experiments performed on different days using separately prepared bacterial cultures. For each independent biological replicate, biofilm-associated cells were collected and stabilized as described above, except that cells recovered from six technical replicate wells per condition were pooled to increase RNA yield and minimize well-to-well variability (Sousa et al., 2014). Each resulting pool represented a single biological replicate.

Total RNA was extracted using the RNeasy^®^ Mini Kit (QIAGEN) with on-column DNase digestion (RNase-Free DNase Set, QIAGEN). To ensure complete removal of residual genomic DNA, extracted RNA was additionally treated with the TURBO DNA-free™ Kit (Invitrogen, Waltham, MA, USA) following the manufacturer’s protocol. RNA integrity was initially assessed by agarose gel electrophoresis, and RNA concentration and integrity number (RIN) were subsequently evaluated using an Agilent TapeStation at the Genomics Unit (Centros de Asistencia a la Investigación, CAI), Universidad Complutense de Madrid (Madrid, Spain).

Directional prokaryotic mRNA library preparation and sequencing were performed by Novogene Company Limited (Cambridge, UK). Ribosomal RNA was depleted using the Illumina Ribo-Zero Plus rRNA Depletion kit (Illumina, San Diego, CA, USA), and strand-specific libraries were constructed using the Novogene NGS Stranded RNA Library Prep Set (PT044) (Novogene) following the provider’s standard workflow. Sequencing was carried out on an Illumina NovaSeq X Plus platform using paired-end 150-bp reads (PE150), targeting approximately 5 Gb of data per sample. The provider processed raw reads to remove adapter sequences, poly-N reads and low-quality reads, and delivered cleaned reads as compressed FASTQ files (*.fq.gz*) for downstream analyses.

Read quality was assessed using FastQC (v0.12.1) (Andrews, 2010) and summarized with MultiQC (v1.33) (Ewels et al., 2016), and reads were further trimmed with Trimmomatic (v0.40) (Bolger et al., 2014) to remove residual adapters and low-quality bases. Trimmed reads were mapped with STAR (v2.7.11b) (Dobin et al., 2013) using competitive alignment against concatenated reference genomes consisting of *S. aureus* USA300 FPR3757 (ASM1346v1; RefSeq GCF_000013465.1) plus the corresponding LAB reference genome (*L. fermentum* IFO 3956, ASM1014v1; RefSeq GCF_000010145.1; *L. reuteri* DSM 20016, ASM1682v1; RefSeq GCF_000016825.1; or *L. salivarius* UCC118, ASM892v1; RefSeq GCF_000008925.1). Genome indices were generated from the corresponding RefSeq GTF annotations, and alignments were performed with bacteria-optimized settings (--alignIntronMax 1). Output Binary Alignment/Map (BAM) files were sorted and indexed with SAMtools (v1.23) (Li et al., 2009), and the relative contribution of each species in LAB-exposed SA4 biofilms was estimated from primary alignments using the idxstats command implemented in SAMtools.

Gene-level counts were obtained with featureCounts (v2.1.1) (Liao et al., 2014) in paired-end mode, counting fragments and assigning reads to gene features grouped by gene_id with the corresponding GTF annotation for each concatenated reference genome. Differential expression analyses were performed in R using DESeq2 (v1.50.2) (Love et al., 2014) after filtering out low-count genes (sum across samples < 10). Models were fitted using the design formula ~ replicate + condition, and *p*-values were adjusted using the Benjamini-Hochberg procedure. Differential expression was assessed separately for each species by comparing LAB-exposed SA4 biofilms with their respective controls. Differentially expressed genes (DEGs) were defined as those presenting an absolute log_2_ fold change |log2FC| > 1 and a statistically significant difference between conditions, based on an adjusted *p*-value (False Discovery Rate, FDR) < 0.05.

Variance-stabilized counts were used for exploratory analyses, including principal component analysis (PCA) and sample-to-sample Pearson correlation. DEGs from *S. aureus* were further compared across LAB exposure conditions to identify shared, pair-specific, and strain-specific responses, whereas DEGs from each LAB strain were analyzed separately to characterize LAB transcriptional responses during interaction with *S. aureus* biofilms.

### 2.5 Statistical analysis and data visualization

All statistical analyses and data visualization were performed in R/RStudio (v4.3.1) using the *ggplot2*, *pheatmap*, *VennDiagram* and *corrplot* packages (Chen and Boutros, 2011; Wickham, 2016; Wei and Simko, 2024; Kolde, 2025). Biofilm-associated CFU counts are presented as mean ± standard deviation (SD). Differences in residual *S. aureus* counts after 24 h of LAB exposure were evaluated relative to the 24-h pre-treatment biofilm (baseline) by one-way analysis of variance (ANOVA), followed by Dunnett’s *post hoc* test. For RT-qPCR analyses, results are presented as log_2_ fold change (log2FC) relative to the corresponding MRS-treated control. The distribution of RT-qPCR log2FC values was assessed using the Shapiro-Wilk test to guide data representation. As normality assumptions were met in most cases, results are presented as mean ± SD. For each gene and experimental condition, differences between 4 h and 16 h log2FC values were evaluated using Welch’s *t*-test. Statistical significance was set at *p* < 0.05 for CFU counts and RT-qPCR analyses.

## 3 Results

### 3.1 Active LAB cultures reduce mature *S. aureus* biofilms and progressively dominate LAB-exposed communities

Both *S. aureus* strains formed dense 24-h mature biofilms at baseline, reaching attached populations of 8.10 ± 0.16 and 8.29 ± 0.02 log_10_ CFU/cm^2^ for SA4 and SA55, respectively (**Figure 1A**). Considering the surface area of each well (1.9 cm^2^), these densities corresponded to approximately 2.4 × 10^8^ SA4 and 3.7 × 10^8^ SA55 CFU per well at the time of LAB addition. Since each biofilm was exposed to approximately 10^5^ LAB CFU, the initial LAB-to-*S. aureus* ratios were approximately 1:2,400 for SA4 and 1:3,700 for SA55, representing a numerical disadvantage of approximately 3.4–3.6 log_10_ units for the LAB.

**Figure 1:**
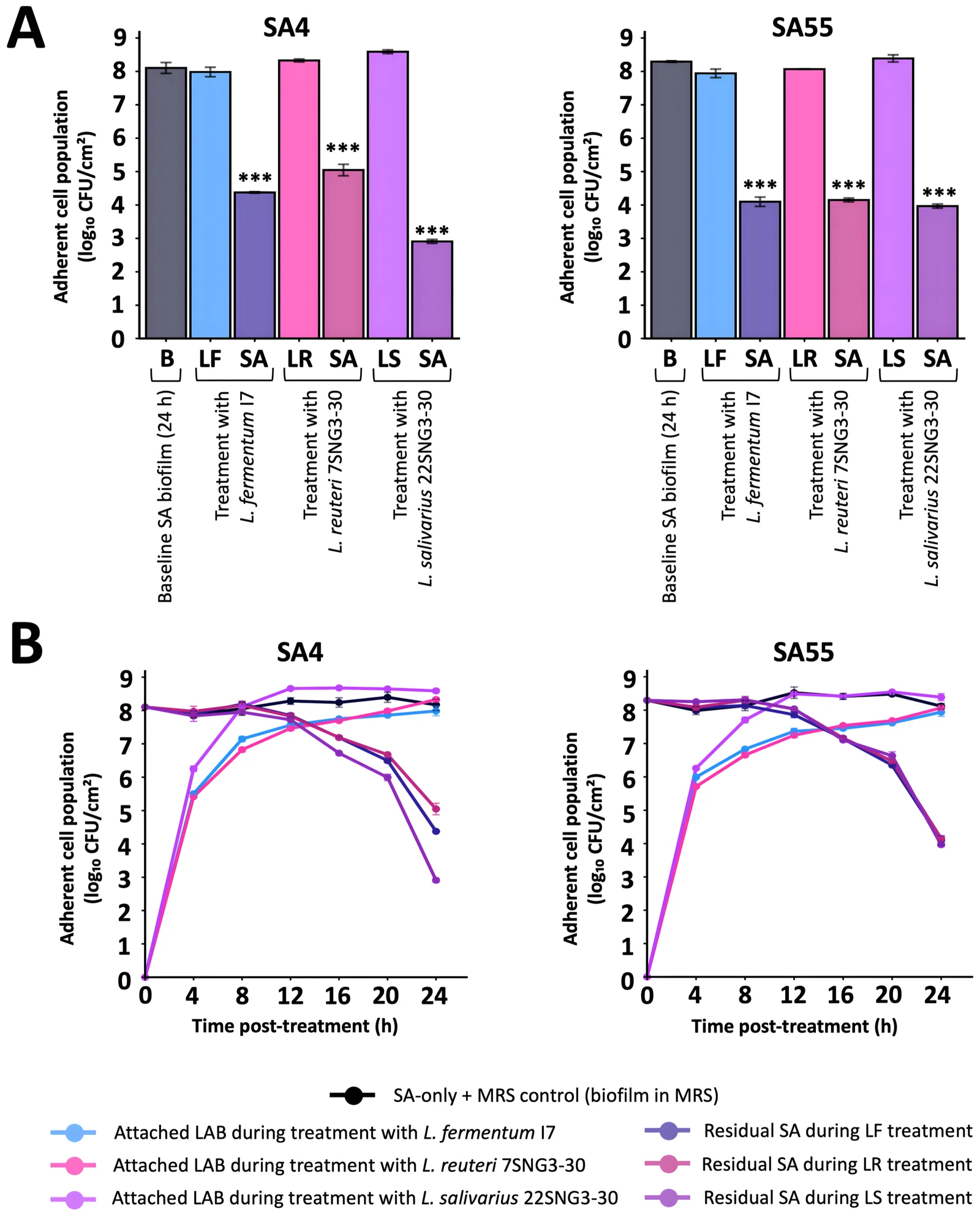
Active LAB cultures destabilize preformed *Staphylococcus aureus* biofilms and progressively dominate LAB-exposed communities. **(A)** Biofilm-associated viable counts (log_10_ CFU/cm^2^) in 24-h preformed biofilms of *S. aureus* SA4 (left) and SA55 (right), measured before treatment (B; baseline pre-treatment biofilm) and after 24 h of exposure to active LAB cultures (*Limosilactobacillus fermentum* I7, LF; *Limosilactobacillus reuteri* 7SNG3-30, LR; or *Ligilactobacillus salivarius* 22SNG3-30, LS). For each LAB treatment, both the attached LAB population (LF/LR/LS) and the residual attached *S. aureus* population (SA) within the treated biofilm were quantified. Bars represent mean ± SD (n = 3). Asterisks indicate significant differences in residual *S. aureus* counts relative to the baseline pre-treatment biofilm (one-way ANOVA followed by Dunnett’s *post hoc* test; ***p < 0.001). **(B)** Temporal dynamics of biofilm-associated viable counts during LAB exposure. Preformed 24-h biofilms of SA4 (left) and SA55 (right) were incubated with active LAB cultures and attached viable counts of LAB (LF/LR/LS) and residual *S. aureus* were determined every 4 h over a 24-h exposure period (0–24 h). The SA + MRS condition (preformed *S. aureus* biofilms incubated with MRS alone) is shown as the time-course control. Data represent mean ± SD (n = 3).

The viable attached *S. aureus* population was markedly reduced after LAB exposure for 24 h (**Figure 1A**). In the case of *S. aureus* SA4, the magnitude of the reduction depended on the LAB strain. Concretely, *L. fermentum* I7 reduced SA4 population to 4.38 ± 0.02 log_10_ CFU/cm^2^ (≈ 3.72-log_10_ reduction from baseline), *L. reuteri* 7SNG3–30 to 5.05 ± 0.17 log_10_ CFU/cm^2^ (≈ 3.05-log_10_ reduction), and *L. salivarius* 22SNG3–30 to 2.91 ± 0.06 log_10_ CFU/cm^2^ (≈ 5.19-log_10_ reduction), being the latter the most effective competitor against SA4 mature biofilms. *S. aureus* SA55 biofilms were also sharply reduced upon the treatment but the effect was similar for the three LAB strains, reaching final cell counts of 4.10 ± 0.14, 4.15 ± 0.06, and 3.97 ± 0.06 log_10_ CFU/cm^2^ after exposure to *L. fermentum* I7, *L. reuteri* 7SNG3-30, and *L. salivarius* 22SNG3-30, respectively (≈ 4.14–4.32-log_10_ reductions). In parallel with the decline in *S. aureus* attached population, LAB colonized the preformed biofilms, reaching high attached populations after 24 h (**Figure 1A**). LAB populations reached 7.98 ± 0.14, 8.33 ± 0.05 and 8.59 ± 0.06 log_10_ CFU/cm^2^ for *L. fermentum* I7, *L. reuteri* 7SNG3–30 and *L. salivarius* 22SNG3-30, respectively, in *S. aureus* SA4 biofilms. Similar LAB counts were observed when SA55 biofilms were used as the target. Consequently, LAB counts exceeded the residual attached *S. aureus* population by approximately 3.28 to 5.68 log_10_ units depending on the LAB strain added to the biofilm (**Figure 1A**).

To characterize the kinetics of competition and to select sampling points for transcriptomic profiling, biofilm-associated counts were monitored every 4 h during the LAB-exposure period (**Figure 1B**). In the control condition (SA + MRS), the attached *S. aureus* population remained stable at high levels (≈8–9 log_10_ CFU/cm^2^) throughout the time tested. During the first 4 h of LAB exposure, biofilm-associated LAB populations increased, reaching cellular densities comparable to those of the *S. aureus* strains. From 12–16 h onwards, the attached *S. aureus* population started to decrease while LAB populations remained high; finally, the largest reductions in biofilm-associated *S. aureus* counts were detected at the 24-h endpoint (**Figure 1B**). The kinetics supported the selection of 4 h and 16 h post-treatment for downstream transcriptional profiling, representing (i) an early interaction stage in which both species are abundant and biofilm biomass remains high, and (ii) a transition stage associated with the onset of *S. aureus* displacement while still providing sufficient biofilm-associated cells for robust RNA extraction and gene expression analyses.

### 3.2 LAB exposure induces a time-dependent transcriptional response in *S. aureus* biofilm cells

To investigate whether LAB-driven displacement of *S. aureus* from mature biofilms was accompanied by changes in virulence- and persistence-related pathways, the expression of a panel of genes involved in quorum sensing/virulence regulation (*agrB, sarA*), secreted effectors/toxins (*hld, psmβ1, seg*), proteolysis and secretion (*sspA, esaA*), biofilm formation/remodeling (*icaA, atl*), iron-related functions (*sfaA*), and stress/proteostasis responses (*sodA, uspA, clpP*) were quantified by RT-qPCR at 4 h and 16 h after LAB exposure in biofilms formed by SA4 and SA55 (**Figure 2**).

**Figure 2:**
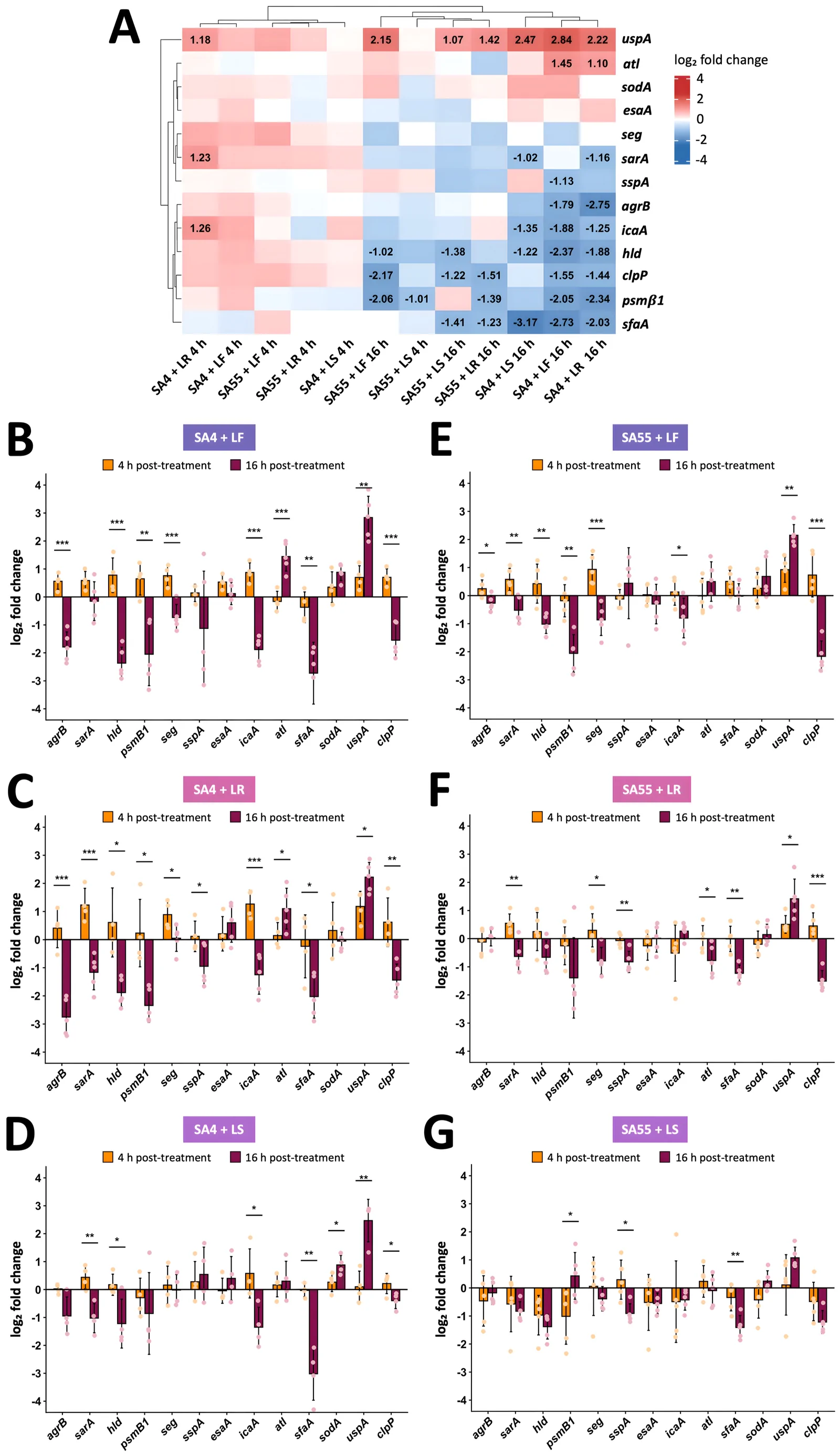
LAB exposure elicits time-dependent transcriptional changes in *Staphylococcus aureus* biofilm cells. RT-qPCR was used to quantify the expression of selected *S. aureus* genes in 24-h preformed biofilms exposed to active LAB cultures (*Limosilactobacillus fermentum* I7, LF; *Limosilactobacillus reuteri* 7SNG3-30, LR; or *Ligilactobacillus salivarius* 22SNG3-30, LS) at 4 h and 16 h post-treatment. Expression changes are shown as log_2_ fold change (log2FC) relative to the corresponding MRS-treated control at each time point, after normalization to the geometric mean of the reference genes *16S rRNA* and *gyrB*. **(A)** Heatmap summarizing log2FC values; only genes meeting the significance and threshold criteria (|log2FC| > 1) are annotated with numerical values. **(B–D)** SA4 biofilms exposed to *L. fermentum* I7 **(B)**, *L. reuteri* 7SNG3-30 **(C)** or *L. salivarius* 22SNG3-30 **(D)**. **(E–G)** SA55 biofilms exposed to *L. fermentum* I7 **(E)**, *L. reuteri* 7SNG3-30 **(F)** or *L. salivarius* 22SNG3-30 **(G)**. Bar plots show mean ± standard deviation (SD) log2FC across independent biological replicates, with individual replicate values overlaid. The number of biological replicates varied by condition: SA4 + LF and SA4 + LR (n = 4 at 4 h; n = 5 at 16 h), SA4 + LS (n = 4), and SA55 biofilms exposed to LAB (n = 5). Orange and purple bars correspond to 4 h and 16 h post-treatment, respectively. Asterisks indicate significant differences between 4 h and 16 h log2FC values for each gene within the same condition (Welch’s t-test; *p < 0.05, **p < 0.01, ***p < 0.001).

There was a time-associated pattern in the *S. aureus* response between 4 and 16 h since most profiles corresponding to early LAB exposures (4 h) grouped, whereas all 16-h profiles clustered within a second major branch, together with the *S. aureus* SA55-*L. salivarius* 4 h challenge (**Figure 2A**). After 4 h of exposure, the transcriptional response was relatively moderate, with a relatively low number of genes exceeding the |log2FC| > 1 threshold. However, prolonged LAB treatment elicited a more extensive transcriptional reprogramming, characterized by stronger differential expression and broader dysregulation across multiple functional modules. This temporal pattern was aligned with the observed differences in CFU counts (**Figure 1B**).

The expression of most of the genes of *S. aureus* SA4 and SA55 remained close to baseline levels after exposure to the LAB strains for 4 h. The main exception was SA4 biofilms exposed to *L. reuteri* 7SNG3-30, where *sarA*, *icaA* and *uspA* were induced (log2FC = 1.23, 1.26 and 1.18, respectively; **Figure 2C**). In SA55 biofilms, no major transcriptional changes were detected at this stage, except for the repression of *psmβ1* after exposure to *L. salivarius* 22SNG3-30 (log2FC = −1.01; **Figure 2G**).

In contrast, pronounced changes were observed in the transcriptional profiles of both *S. aureus* strains after being exposed to the LAB strains for 16 h (**Figures 2B–G**). Expression of *uspA* increased in all *S. aureus*-LAB combinations (log2FC = 1.07–2.84). Exposure of *S. aureus* SA4 to *L. fermentum* I7 and *L. reuteri* 7SNG3–30 also induced the expression of *atl*, with mean log2FC values of 1.45 and 1.10, respectively. In contrast, several virulence- and interaction-associated genes were repressed in both *S. aureus* strains, although the level of repression was higher in SA4 than in SA55. When SA4 biofilms were exposed to the LAB strains, there was a downregulation of the following genes: *sfaA* (−2.03 to −3.17), *agrB* (−1.79 to −2.75), *icaA* (−1.25 to −1.88), *hld* (−1.22 to −2.37) and *psmβ1* (−2.05 to −2.34). In the case of SA55, a similar but, overall, weaker response was detected for several genes, including *sfaA* (−1.23 to −1.41), *hld* (−1.02 to −1.38), *psmβ1* (−1.01 to −2.06) and *clpP* (−1.22 to −2.17).

### 3.3 RNA-seq analysis of *S. aureus* SA4 biofilms exposed to LAB reveals extensive transcriptional remodeling

Based on the RT-qPCR results, which showed a broader transcriptional response at 16 h in both strains but more pronounced and consistent changes across the three LAB treatments in SA4, RNA-seq was performed on SA4 biofilms harvested after 16 h of LAB exposure. In relation to the monospecies control biofilms, SA4 libraries (22.9–33.6 million mapped reads) aligned almost exclusively to the *S. aureus* reference genome (≥99.95% across replicates), while LAB libraries mapped predominantly to their cognate references, with 25.6–26.4 million mapped reads for *L. fermentum* I7 (98.61–99.32%), 24.1–38.3 million for *L. reuteri* 7SNG3-30 (99.98–99.999%), and 29.4–31.4 million for *L. salivarius* 22SNG3-30 (99.993–99.999%). These results support robust species assignment, with no evidence of sample cross-contamination or systematic misclassification of reads.

In SA4 biofilms exposed to LAB for 16 h, the distribution of mapped reads differed markedly depending on the LAB strain (**Table 2**). Exposure to *L. fermentum* I7 resulted in 24.66–63.74 million mapped reads and the percentage of *S. aureus* reads (89.25–95.39%) was notably higher than that of *L. fermentum* reads (4.61–10.75%). Exposure of this staphylococcal strain to *L. reuteri* 7SNG3–30 resulted in 28.58–32.81 million mapped reads although a more balanced distribution of the percentages was observed (*S. aureus:* 35.27–50.33%; *L. reuteri:* 49.67–64.73%). SA4 biofilms exposed to *L. salivarius* 22SNG3–30 yielded 31.86–33.49 million mapped reads but most of them were assigned to the LAB strain (79.72–83.86%).

**Table 2:** Relative distribution of mapped reads assigned to *Staphylococcus aureus* and lactic acid bacteria (LAB) reference genomes in LAB-exposed SA4 biofilms after 16 h of interaction.

Condition (LAB exposure)	Replicate	Total mapped reads (M)	*S. aureus* mapped reads (M)	*S. aureus* (%)	LAB mapped reads (M)	LAB (%)
SA4 exposed to *L. fermentum* I7	1	24.66	23.53	95.39	1.14	4.61
	2	37.36	34.11	91.30	3.25	8.70
	3	63.74	56.89	89.25	6.85	10.75
SA4 exposed to *L. reuteri* 7SNG3-30	1	28.58	14.39	50.33	14.20	49.67
	2	30.79	12.74	41.38	18.05	58.62
	3	32.81	11.57	35.27	21.24	64.73
SA4 exposed to *L. salivarius* 22SNG3-30	1	31.86	5.92	18.59	25.93	81.41
	2	32.84	6.66	20.28	26.18	79.72
	3	33.49	5.40	16.14	28.09	83.86

Reads were competitively mapped against concatenated reference genomes consisting of *S. aureus* USA300 FPR3757 and the corresponding LAB reference genome: *Limosilactobacillus fermentum* IFO 3956, *Limosilactobacillus reuteri* DSM 20016 or *Ligilactobacillus salivarius* UCC118, depending on the mixed-species condition.

Exploratory analysis of variance-stabilized counts further supported the robustness of the RNA-seq dataset. Principal component analysis and sample-to-sample Pearson correlation clustering showed a clear grouping of the samples into SA4 controls, LAB monospecies biofilms and LAB-exposed SA4 biofilms (**Figure 3**). Together, PC1 and PC2 accounted for 85.59% of the total variance. PC1 (51.56%) appeared to reflect primarily differences in species composition, with the *L. reuteri* and *L. salivarius* monospecies controls located at opposite extremes and the mixed biofilms occupying more intermediate positions. PC2 (34.03%) further contributed to distinguishing the monospecies controls from the mixed-species interactions, although this separation was dependent on the LAB strain. Biofilms exposed to *L. fermentum* I7 clustered close to the SA4 controls, in agreement with the predominance of *S. aureus* transcripts in this strain combination.

**Figure 3:**
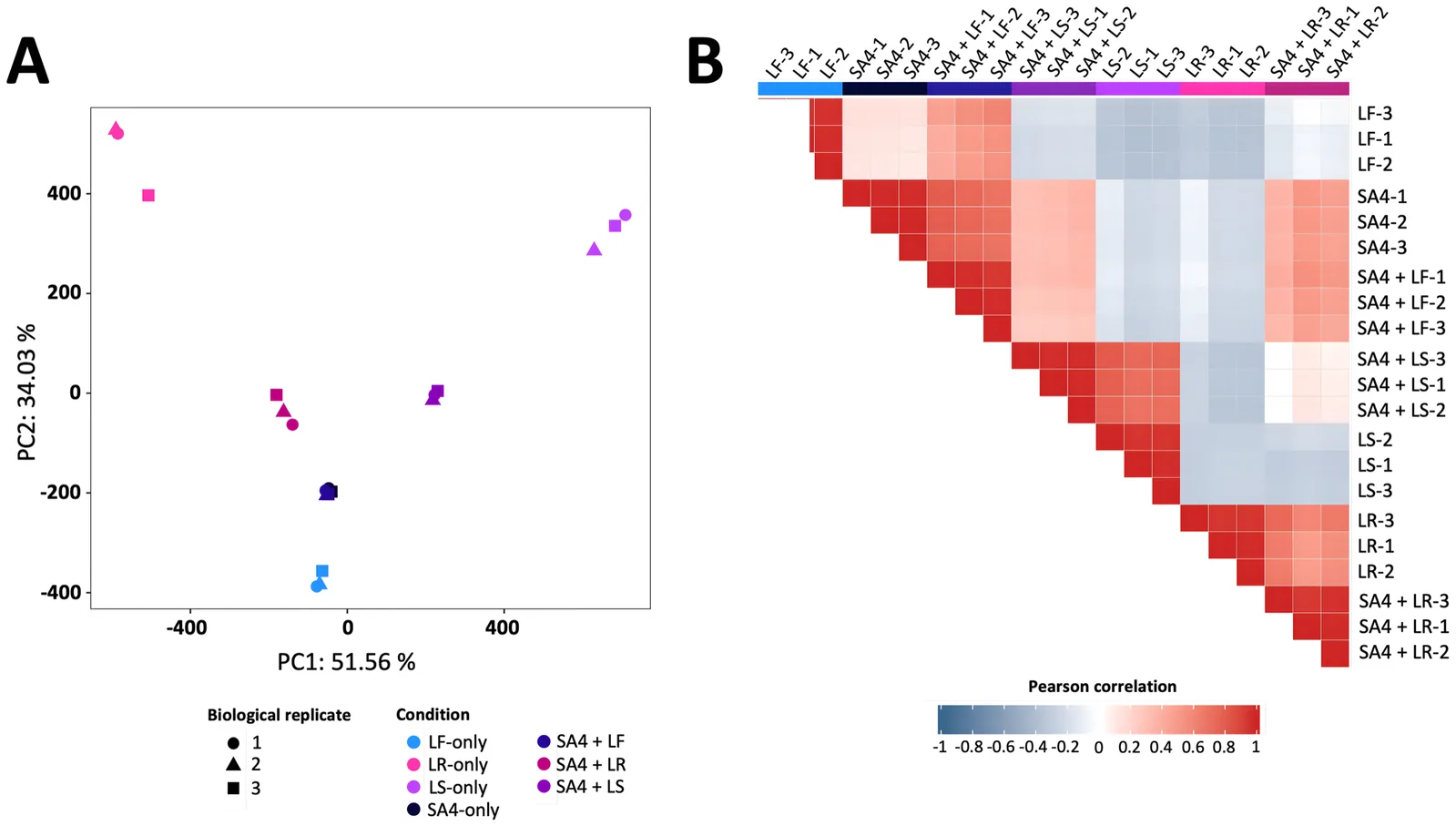
Global transcriptomic relationships among *Staphylococcus aureus* SA4 control biofilms, single-species LAB biofilms and LAB-exposed SA4 biofilms at 16 h post-treatment. **(A)** Principal component analysis (PCA) of variance-stabilized RNA-seq counts showing the distribution of SA4 control biofilms exposed to MRS alone, single-species LAB biofilms, and preformed SA4 biofilms exposed to LAB for 16 h. Each point represents an individual biological replicate; colors indicate experimental conditions, and point shapes indicate the biological replicate number. **(B)** Pearson correlation heatmap between individual RNA-seq libraries based on variance-stabilized counts. Rows and columns correspond to individual biological replicates, with sample labels indicating the experimental condition and replicate number. The color scale represents Pearson correlation coefficients, and hierarchical clustering groups samples according to global transcriptomic similarity. SA4, *Staphylococcus aureus* SA4 control biofilms; LF, *Limosilactobacillus fermentum* I7 biofilms; LR, *Limosilactobacillus reuteri* 7SNG3–30 biofilms; LS, *Ligilactobacillus salivarius* 22SNG3–30 biofilms; SA4 + LF, *S. aureus* SA4 biofilms exposed to *L. fermentum* I7; SA4 + LR, *S. aureus* SA4 biofilms exposed to *L. reuteri* 7SNG3-30; SA4 + LS, *S. aureus* SA4 biofilms exposed to *L. salivarius* 22SNG3-30.

Volcano plots for each LAB exposure condition revealed extensive transcriptional remodeling in *S. aureus* biofilm cells after exposure to the LAB strains (**Figure 4**). In SA4 biofilm cells, 776, 645 and 585 genes were upregulated after 16 h exposure to *L. fermentum* I7, *L. reuteri* 7SNG3–30 and *L. salivarius* 22SNG3-30, respectively, while 821, 712 and 716 genes were downregulated, respectively. In contrast, the transcriptomic response of the LAB strains was less intense and strain-dependent, with 103 upregulated and 151 downregulated genes in *L. fermentum* I7, 178 upregulated and 145 downregulated genes in *L. reuteri* 7SNG3-30, and 19 upregulated and 39 downregulated genes in *L. salivarius* 22SNG3-30 (**Figure 4**).

**Figure 4:**
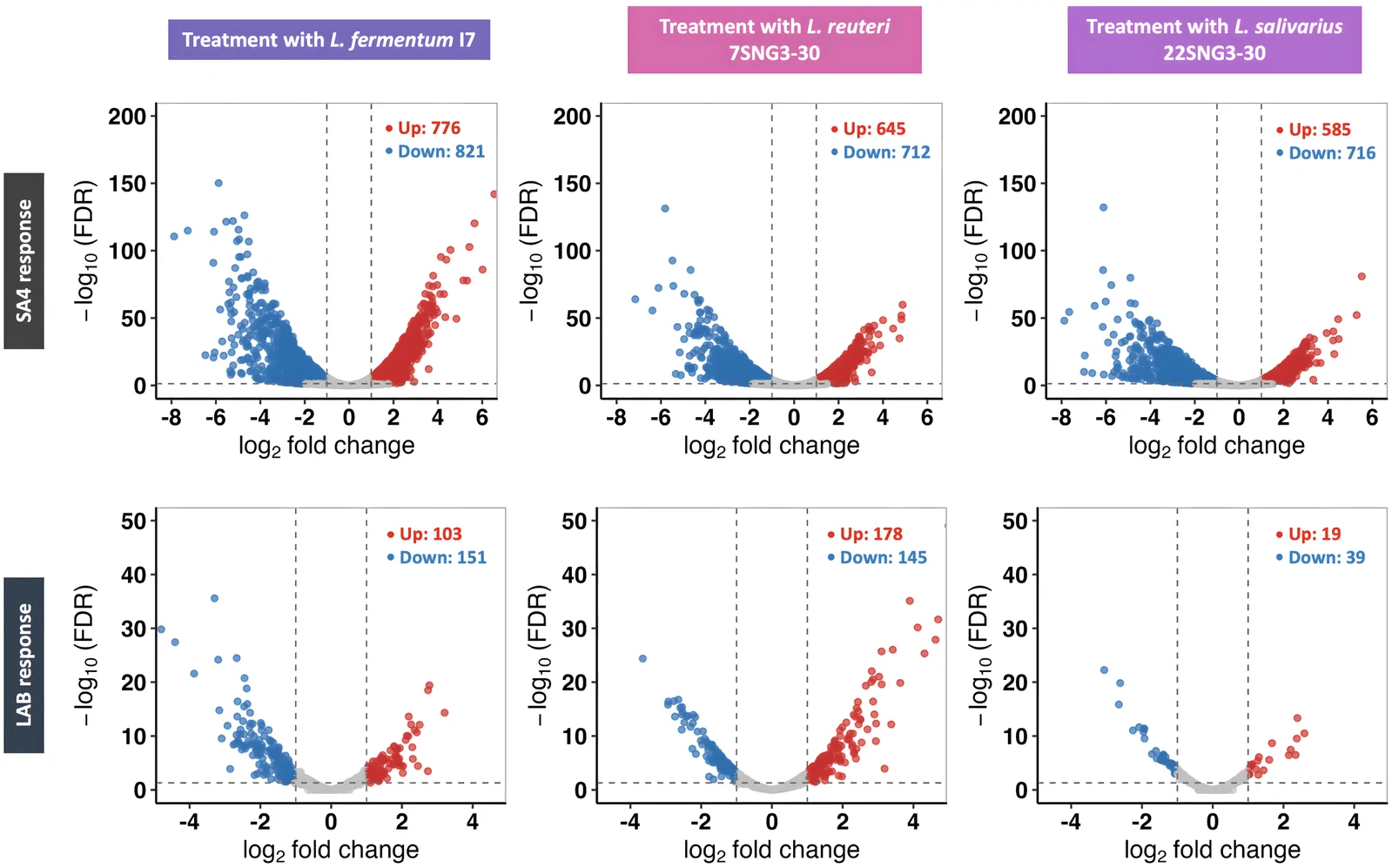
Differential gene expression in *Staphylococcus aureus* SA4 and LAB biofilms after 16 h of LAB exposure. Volcano plots showing significantly upregulated and downregulated genes in *S. aureus* SA4 (top row) and the corresponding lactic acid bacteria (LAB) strain (bottom row) after 16 h of exposure under each experimental condition. Columns correspond to exposure of preformed *S. aureus* SA4 biofilms to *Limosilactobacillus fermentum* I7 (LF), *Limosilactobacillus reuteri* 7SNG3-30 (LR) and *Ligilactobacillus salivarius* 22SNG3-30 (LS), together with the corresponding LAB biofilm responses under the same conditions. Differential expression was calculated relative to the respective single species biofilm controls (SA4 control biofilms exposed to MRS alone for *S. aureus*; LF, LR and LS biofilms for LAB). Each point represents an individual gene. The *x*-axis indicates log_2_ fold change and the *y*-axis shows -log_10_ adjusted *p*-value (false discovery rate, FDR). Genes meeting the significance thresholds (FDR < 0.05 and |log2FC| > 1) are highlighted, with upregulated genes shown in red, downregulated genes in blue, and non-significant genes in grey. The number of upregulated and downregulated genes detected in each comparison is indicated in the upper right corner of each panel.

### 3.4 Shared transcriptional responses of *S. aureus* to LAB exposure

Differentially expressed genes were examined using a heatmap of log2FC values together with Venn diagrams of upregulated and downregulated genes across the three LAB treatments to assess whether the extent of the transcriptional response of *S. aureus* to LAB was similar or different depending on the LAB strain (**Figures 5A–C**). The heatmap includes all *S. aureus* genes significantly regulated in at least one experimental condition (FDR < 0.05 and |log2FC| > 1; n = 1791; **Supplementary Table 1**). It revealed broad clusters of co-regulated genes with similar expression patterns across treatments, alongside smaller subsets showing condition-dependent regulation (**Figure 5A**). Consistent with this pattern, the Venn diagrams (**Figures 5B, C**) showed a substantial *core* of genes regulated in response to *L. fermentum* I7, *L. reuteri* 7SNG3–30 and *L. salivarius* 22SNG3-30, comprising 588 consistently downregulated genes and 444 consistently upregulated genes (**Supplementary Table 1**). Changes in the expression of some genes were only found after exposure to one or two LAB strains, indicating that the *S. aureus* SA4 response to LAB combined a strong conserved transcriptional pattern with some strain-dependent features (see Section 3.5).

**Figure 5:**
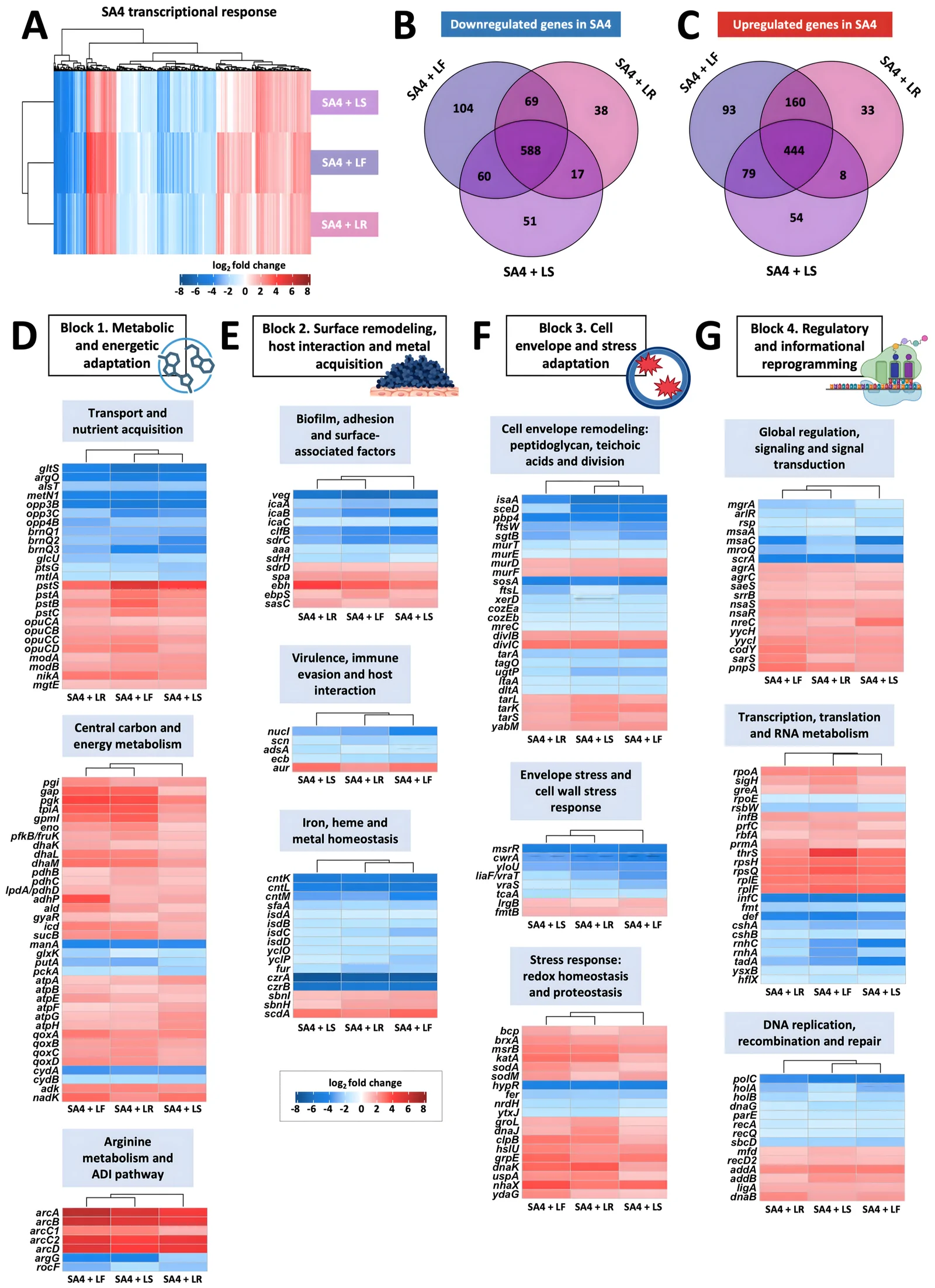
Shared transcriptional reprogramming of *Staphylococcus aureus* SA4 in response to LAB exposure. **(A)** Heatmap showing log_2_ fold change (log2FC) values for *S. aureus* SA4 genes in preformed *S. aureus* SA4 biofilms after 16 h exposure to *Limosilactobacillus fermentum* I7 (LF), *Limosilactobacillus reuteri* 7SNG3-30 (LR) or *Ligilactobacillus salivarius* 22SNG3-30 (LS), relative to *S. aureus* SA4 control biofilms exposed to MRS alone. Genes with significant differential expression in at least one condition (FDR < 0.05 and |log2FC| > 1; n = 1,791 genes) are shown. Colours indicate log2FC values, with red denoting upregulation and blue denoting downregulation. Genes and conditions were hierarchically clustered according to their transcriptional profiles across treatments. **(B)** Venn diagram showing the overlap of downregulated *S. aureus* SA4 genes across the three LAB exposure conditions. **(C)** Venn diagram showing the overlap of upregulated *S. aureus* SA4 genes across the three LAB exposure conditions. **(D–G)** Heatmaps showing *S. aureus* SA4 genes shared across the three LAB exposure conditions, organized into four major functional blocks derived from the integrated transcriptional response: metabolic and energetic adaptation **(D)**, surface remodeling, host interaction and metal acquisition **(E)**, cell envelope and stress adaptation **(F)**, and regulatory and informational reprogramming **(G)**. In heatmaps, rows represent genes and columns represent the three LAB exposure conditions. Colours indicate log2FC values, with red denoting upregulation and blue denoting downregulation.

Selected subsets of the *core* regulated genes were organized into four functional modules (**Figures 5D–G**). The first module included genes involved in transport, central carbon metabolism, energy conservation and arginine catabolism processes (**Figure 5D**). Genes encoding transport systems for amino acids, peptides and preferred carbon sources (*gltS, argO, alsT, metN1, opp3B, opp3C, opp4B, brnQ1, brnQ2, brnQ3, glcU, ptsG* and *mtlA*) were consistently repressed, whereas genes implicated in phosphate transport (*pstS, pstA, pstB*, *pstC)* and osmoprotection or ion-homeostasis (*opuCA-opuCD, modA, modB, nikA*, *mgtE)* were induced. In parallel, glycolytic and carbon-processing genes (*pgi, gap, pgk, tpiA, gpmI, eno, pfkB/fruK* and *dhaK-dhaL-dhaM*) were upregulated, together with genes linked to pyruvate processing and connected intermediary metabolism (*pdhB, pdhC, lpdA/pdhD, adhP, ald, gyaR, icd* and *sucB*), while some genes related to the utilization of alternative carbon and nitrogen sources (*manA*, *glxK*, *putA*) and the gluconeogenic gene *pckA* were repressed. Respiratory and energy-conserving functions were also modulated, with induction of *atpA, atpB, atpE, atpF, atpG, atpH* and the *qoxABCD* operon, and repression of *cydA* and *cydB*; in addition, *adk* and *nadK* were upregulated, indicating changes in adenylate and redox cofactor metabolism. The arginine deiminase pathway, strongly associated with adaptation to acidic environments, was also strongly activated, with marked upregulation of *arcA, arcB, arcC1, arcC2* and *arcD*, together with repression of *argG* and *rocF*, two genes associated with arginine biosynthesis and catabolism.

A second shared module involved surface-associated, virulence-related and metal homeostasis functions (**Figure 5E**). Within the surface-associated category, genes involved in poly-β-(1,6)-N-acetylglucosamine/polysaccharide intercellular adhesin (PNAG/PIA)-dependent biofilm matrix production and canonical adhesion (*veg, icaA, icaB, icaC, clfB, sdrC*, *aaa* and *sdrH*) were consistently repressed, whereas a smaller subset of genes encoding surface-associated adhesins and factors involved in host interaction, immune evasion or biofilm formation (*sdrD, spa, ebh, ebpS* and *sasC*) was upregulated. Similarly, several immune evasion or host-associated genes (*nucI, scn, adsA* and *ecb*) were repressed, while the gene *aur*, encoding the secreted metalloprotease aureolysin, was induced in the three conditions. Genes involved in iron, heme and metal homeostasis were also broadly affected. Concretely, LAB treatments induced the repression of the staphylopine biosynthetic genes *(cntK, cntL* and *cntM*), the staphyloferrin A export gene *sfaA*, heme uptake genes (*isdA, isdB, isdC* and *isdD)*, ferric-chelate transport-related genes (*yclO* and *yclP)*, and metal-homeostasis-related genes (*fur, czrA* and *czrB*). In parallel, *sbnI* and *sbnH*, involved in staphyloferrin B biosynthesis and regulation, were upregulated, together with *scdA*, a gene associated with an iron-sulfur repair cluster.

A third shared module comprised genes related to cell envelope biogenesis, cell division and stress adaptation (**Figure 5F**). Multiple genes involved in peptidoglycan remodelling and maturation (*isaA, sceD, pbp4, ftsW, sgtB, murT* and *murE*) were downregulated, whereas *murD* and *murF* were induced. A repressive response was also observed among genes associated with cell division and morphogenesis (s*osA, ftsL, xerD, cozEa, cozEb* and *mreC*), genes involved in teichoic acid and lipoteichoic acid biosynthesis and modification (*tarA, tagO, ugtP, ltaA* and *dltA*), and genes associated with envelope and cell wall stress responses (*msrR, cwrA, yloU, liaF/vraT, vraS* and *tcaA*). Moreover, a broad stress-related response was observed, with induction of antioxidant and thiol-redox genes (*bcp, brxA, msrB, katA, sodA* and *sodM*), upregulation of chaperone- and proteostasis-associated genes (*groL, dnaJ, clpB, hslU, grpE* and *dnaK*), and differential regulation of general stress-associated genes (*uspA, nhaX*, *ydaG, hypR, fer, nrdH* and *ytxJ*).

Finally, within the fourth shared module including genes participating in cell reprogramming functions, several genes linked to colonization, virulence or biofilm-associated behaviour (*mgrA, arlR, rsp, msaA, msaC, mroQ* and *scrA*) were consistently downregulated, whereas adaptive signaling components and regulators (*agrA, agrC, saeS, srrB, nsaS, nsaR, nreC, yycH, yycI, codY, sarS* and *pnpS*) were induced (**Figure 5G**). This was accompanied by changes in transcription-, translation- and RNA metabolism-related genes, including induction of *rpoA, sigH, greA, infB, prfC, rbfA, prmA, thrS, rpsH, rpsQ, rplE* and *rplF*, together with repression of *rpoE, rsbW, infC, fmt, def, cshA, cshB, rnhC, rnhA, tadA, ysxB* and *hflX*. Genes related to DNA replication and recombination were predominantly downregulated. By contrast, a distinct subset of genes associated with DNA maintenance and repair was consistently upregulated (*mfd, recD2, addA, addB*, *ligA* and *dnaB*).

### 3.5 Pair-specific and strain-specific transcriptional signatures in *S. aureus* SA4 in response to individual LAB strains

Despite the strongly conserved *core* response described above, some additional transcriptional changes were detected, revealing LAB strain-specific responses of S. *aureus* SA4 biofilm cells (**Supplementary Table 1**). Shared transcriptional changes induced by the exposure to *L. fermentum* I7 and *L. reuteri* 7SNG3–30 were dominated by upregulated genes (*secA2, asp3, gtf1, gtf2, csbB*, *sasF*, *capE/capF*, *atl*, *lip2*, *esxA*, *sspB/sspC*) whereas *hlgA* was repressed. This shared response was also characterized by the upregulation of genes linked to alternative carbohydrate uptake, pyruvate/redox metabolism and pH/ion homeostasis, and notably, it also included the induction of several phage- and mobile-element-associated genes. In contrast, the transcriptional changes shared by *L. fermentum* I7 and *L. salivarius* 22SNG3–30 addressed carbohydrate and central carbon metabolism genes, together with coordinated repression of ion-homeostasis functions, and induction of selected envelope- and secretion-associated genes (*femX, galU, tarI, tagE3/sdgA, essA* and *essB*). Few transcriptional changes were shared by *L. reuteri* 7SNG3–30 and *L. salivarius* 22SNG3-30, and they were mostly associated with the repression of genes related to envelope-related functions and induction of the cell-wall sensor kinase (*vicK/walk)*, the heme-homeostasis transporter component (*hrtA)*, the type VII secretion gene (*esaA)*, and the energy-related genes (*atpD* and *pdhA)*.

Apart from the transcriptional responses shared by two or three of the LAB strains tested in this work, *S. aureus* SA4 biofilm cells also displayed a LAB strain-specific transcriptional response (**Supplementary Table 1**). Genes related to host interaction and adhesion (*eap, FLIPr, sraP/sasA, SSL1*), competence (*comGC, comGD, comGF, comFC*) and nutrient/metal scavenging (*cycA, purC, bioD, sirB, cntC/cntD, feoB, isdH*) were repressed, whereas the expression of a set of genes linked to carbon metabolism and regulatory adaptation (*glcK, gpmA, ptsH, lacG, fbp, lctP, panC, panD, ribF, fhs, sarR, sarX* and *luxS*) was induced in response to the presence of *L. fermentum* I7. The transcriptional response towards *L. reuteri* 7SNG3–30 was characterized by repression of selected nucleotide/cofactor- and membrane-associated functions (*pyrR, bioF, ctaB, fabZ* and *tagG*) and induction of genes linked to envelope maintenance and metabolic adjustment (*ponA, glmU, lrgA, ureD, ureG, sdhA, sucD* and *ytsJ*), interaction-related factors (*vwb, sdrE*) and a phenol-soluble modulin (*PSMα4*). Finally, *L. salivarius* 22SNG3–30 triggered a more regulatory and stress-associated response, with repression of several biofilm- and surface-associated genes (*gdpS, femA, cap8D, sarA, sbi, vraX* and *merA*), together with induction of regulatory, redox/proteostasis, DNA-associated and cell-division-related functions (*saeR, sarV, ctsR, hfq, cspA, trxB, recF, guaA, gyrA, ftsA, ftsZ* and *tagH*).

### 3.6 Cross-validation of *S. aureus* SA4 transcriptional responses by RT-qPCR and RNA-seq

Since the RNA-seq analysis was performed on SA4 biofilms collected after 16 h of LAB exposure, cross-validation between the targeted RT-qPCR analysis and the global RNA-seq dataset was restricted to the tested RT-qPCR conditions.

Overall, the results obtained by RT-qPCR and RNA-seq showed a high degree of agreement, especially in those genes showing larger transcriptional changes (**Figure 6**). As an example, *icaA* and *sfaA* were consistently downregulated after LAB exposure in both datasets, supporting the repression of biofilm matrix-associated and iron-related functions in SA4 biofilms. Conversely, *uspA* was strongly induced in the three experimental conditions as assessed by both approaches, while *sodA* also showed an overall positive trend, although its induction was less noticeable by RT-qPCR in the *L. reuteri* 7SNG3–30 treatment. Concordant data were also obtained for *atl*, whereas *sarA* and *esaA* displayed smaller or LAB strain-dependent changes. Despite these quantitative differences, which are expected due to the distinct normalization strategies, dynamic ranges and analytical frameworks of RT-qPCR and RNA-seq, the overall directionality of the transcriptomic response was largely preserved. These results support the robustness of the 16-h transcriptional responses and indicate that the main LAB-associated changes detected by RNA-seq in SA4 biofilm cells were consistent with the targeted RT-qPCR analysis.

**Figure 6:**
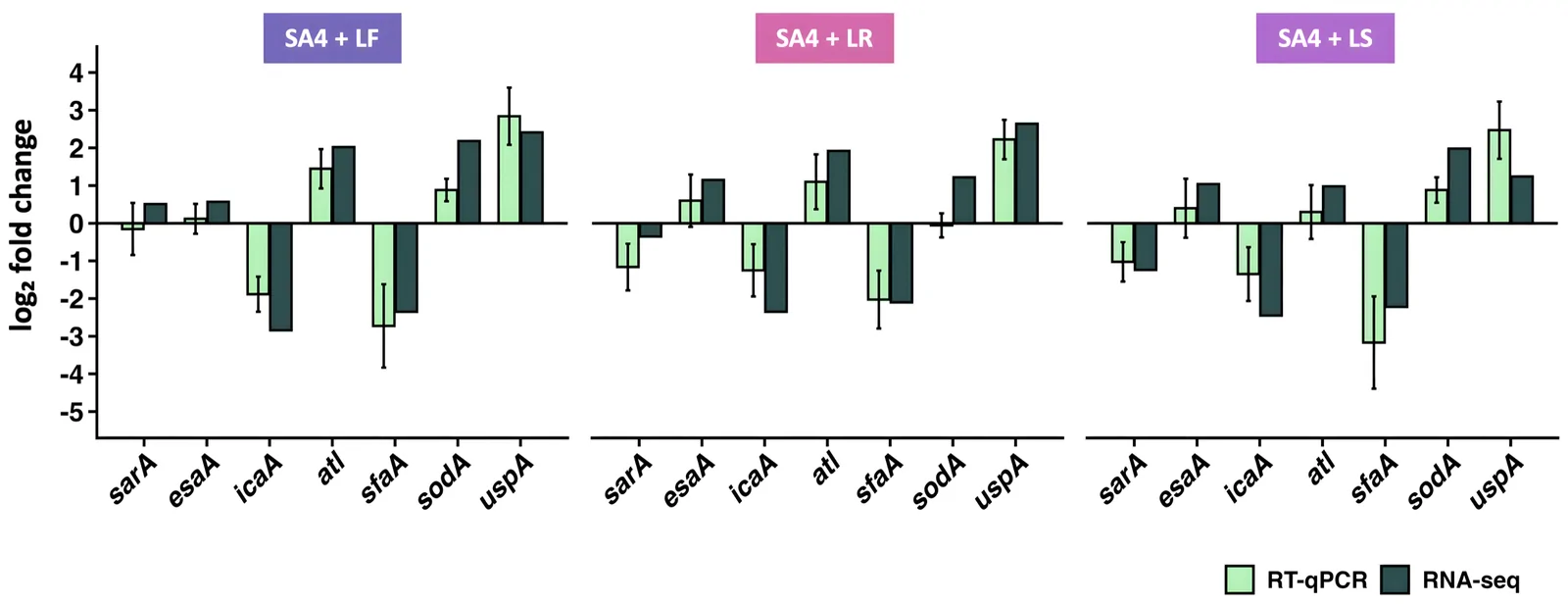
Cross-validation of RNA-seq and RT-qPCR expression changes in *Staphylococcus aureus* SA4 biofilms after 16 h of LAB exposure. Expression changes are shown for selected *S. aureus* genes quantified by RT-qPCR and RNA-seq in SA4 biofilms exposed for 16 h to *Limosilactobacillus fermentum* I7 (LF), *Limosilactobacillus reuteri* 7SNG3-30 (LR) or *Ligilactobacillus salivarius* 22SNG3-30 (LS). RT-qPCR bars represent mean log_2_ fold change ± SD from independent biological replicates (*n* = 5 for SA4 + LF and SA4 + LR; *n* = 4 for SA4 + LS), after normalization to the geometric mean of *16S rRNA* and *gyrB* and relative to the corresponding MRS-treated control. RNA-seq bars represent DESeq2 model-based log2FC estimates obtained from three biological replicates per condition and expressed relative to SA4 control biofilms exposed to MRS alone.

### 3.7 Transcriptomic response of LAB during interaction with *S. aureus* SA4 biofilms

All the LAB strains successfully colonized SA4 biofilms and contributed to *S. aureus* displacement but their transcriptional responses differed substantially among strains and were moderate in comparison to those exhibited by the staphylococcal strain.

In *L. fermentum* I7, upregulated genes were mainly associated with translation, nucleotide-related functions, nutrient transport, surface/envelope adaptation and cofactor/redox-associated processes (**Figure 7A**; **Supplementary Table 2**). This upregulated profile is consistent with translational and biosynthetic activity, together with nutrient acquisition and envelope-associated adaptation during interaction with SA4 biofilms. Downregulated genes in *L. fermentum* I7 indicated repression of several stress-, redox-, nitrogen metabolism- and mobile element-associated modules (**Figure 7A**; **Supplementary Table 2**). Concerning *L. reuteri* 7SNG3-30-SA4 interactions, upregulated genes were mainly associated with nucleotide biosynthesis, nutrient acquisition, translation, surface adaptation and redox-associated metabolism. In contrast, downregulated genes reflected the repression of specific metabolic, regulatory and envelope-associated modules (**Figure 7B**; **Supplementary Table 3**).

**Figure 7:**
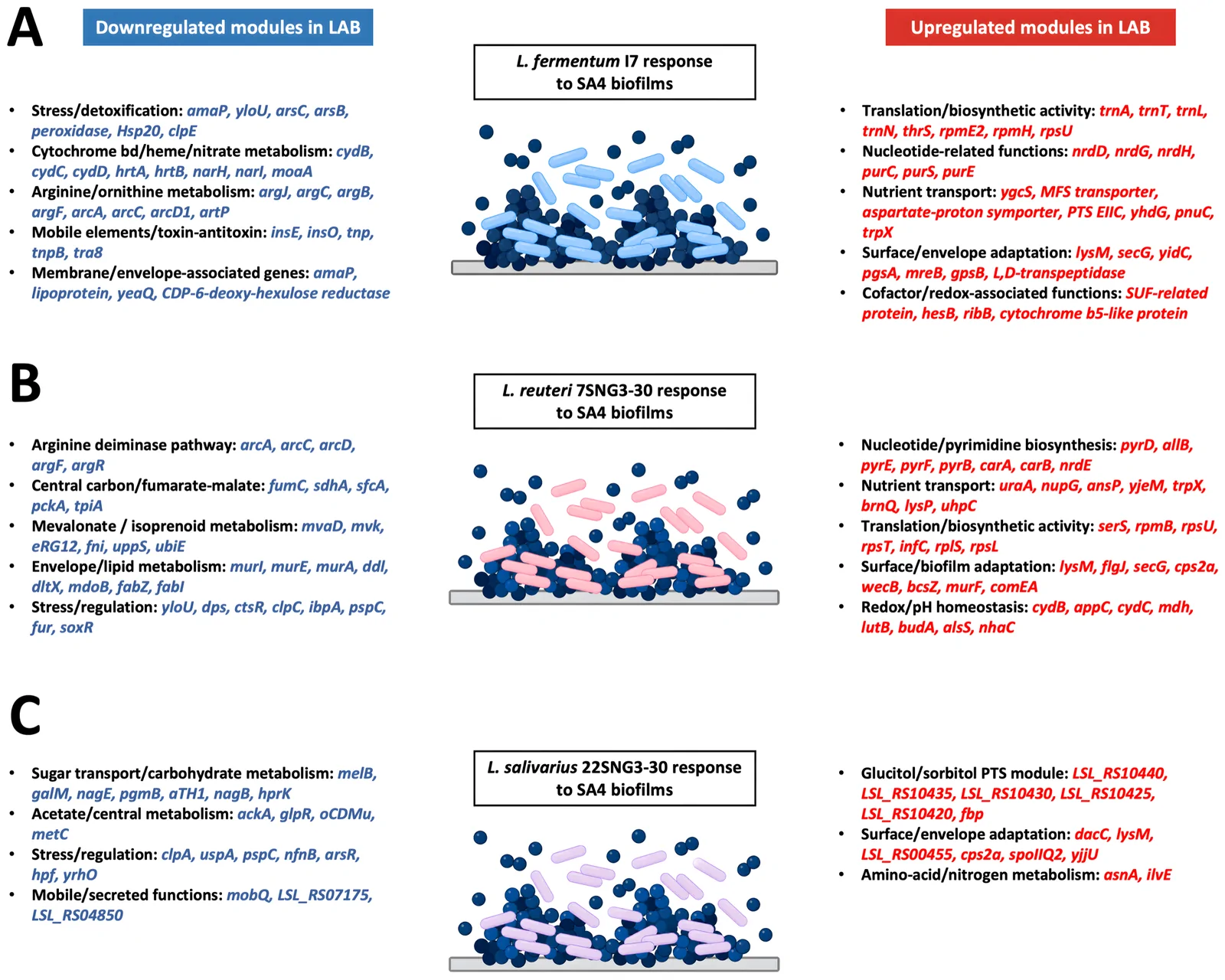
Transcriptomic responses of LAB during interaction with *Staphylococcus aureus* SA4 biofilms. Schematic representation of the main functional modules associated with upregulated and downregulated genes in **(A)** *Limosilactobacillus fermentum* I7, **(B)** *Limosilactobacillus reuteri* 7SNG3–30 and **(C)** *Ligilactobacillus salivarius* 22SNG3–30 during interaction with preformed *S. aureus* SA4 biofilms. Blue indicates downregulated genes and red indicates upregulated genes. Only representative genes from each functional module are shown. Full differential expression results are provided in **Supplementary Tables 2–4**.

In contrast, *L. salivarius* 22SNG3–30 showed a more limited transcriptional response during interaction with SA4 biofilms (**Figure 7C**; **Supplementary Table 4**). Upregulated genes were primarily associated with carbohydrate uptake/metabolism and surface/envelope adaptation. The most induced genes were those related to glucitol/sorbitol transport and regulation (*LSL_RS10440*, *LSL_RS10435*, *LSL_RS10430*, *LSL_RS10425* and *LSL_RS10420*) together with the gene encoding fructose-1,6-bisphosphatase (*fbp*), which is involved in gluconeogenesis. Conversely, downregulated genes included several carbohydrate transport and metabolism genes (*melB*, *galM*, *nagE*, *pgmB*, *aTH1*, *nagB* and *hprK*) together with genes associated with acetate/central and amino-acid metabolism (*ackA*, *glpR*, *oCDMu* and *metC*). Stress-, regulation- and mobile/secreted function-associated genes (*clpA*, *uspA*, *pspC*, *nfnB*, *arsR*, *hpf*, *yrhO*, *mobQ*, *LSL_RS07175* and *LSL_RS04850*) were also repressed. Overall, the transcriptional response of *L. salivarius* 22SNG3–30 was characterized by a remodelling of carbohydrate metabolism and surface/envelope-associated functions, reflecting a more limited differential transcriptional response compared to those of *L. fermentum* I7 and *L. reuteri* 7SNG3-30.

## 4 Discussion

Lactational mastitis remains a clinically relevant condition in which *S. aureus* is particularly important because of its ability to adhere and persist in mammary ducts, forming biofilms and triggering a strong local and systemic inflammatory response (Foster et al., 2014; Angelopoulou et al., 2018). These traits may compromise conventional antibiotic-based therapies, thereby driving the search for alternative or complementary strategies compatible with breastfeeding, including the use of LAB with target-specific probiotic potential (Jiménez et al., 2021; Matera et al., 2025). In this study, active LAB cultures colonized mature *S. aureus* biofilms formed by acute mastitis-associated strains and progressively dominated the biofilm communities, causing substantial reductions in the attached staphylococcal populations. This finding is consistent with our previous work, in which the same LAB strains and their cell-free supernatants disrupted *S. aureus* biofilm structure and reduced biofilm cell viability (Jurado et al., 2025b). It also agrees with a previous study showing that selected *L. fermentum* and *L. salivarius* strains can inhibit *S. aureus* (Kang et al., 2017). The present study extends these phenotypic observations by showing that LAB-mediated biofilm displacement is accompanied by marked transcriptional reprogramming of *S. aureus* biofilm cells during interspecies interaction.

Importantly, the antibiofilm effect observed did not follow an immediate killing-like pattern. LAB populations increased rapidly within the preformed biofilm during the first hours of exposure, whereas the decline in attached *S. aureus* counts became evident later, particularly around 16 h. This temporal offset suggests that LAB-mediated destabilization of mature *S. aureus* biofilms occurs as a progressive competitive transition rather than as an immediate bactericidal action. LAB antagonism against *S. aureus* is a multifactorial and context-dependent phenomenon involving nutritional competition, secreted inhibitory compounds, direct bacterial interactions and changes in surface-, matrix- and metabolism-associated traits (Charlier et al., 2009; Kang et al., 2017; Mao et al., 2023). The RT-qPCR data supported this interpretation by showing that the transcriptional response was also time-dependent.

RT-qPCR analysis showed that LAB exposure for 16 h was associated with repression of several staphylococcal genes linked to quorum sensing, virulence, biofilm matrix production and iron acquisition, together with consistent induction of the stress/adaptation marker *uspA*. The downregulation of *agrB* and of the *agr*/RNAIII-associated effector genes *hld* and *psmβ1* is particularly relevant because interference with *S. aureus* quorum sensing has been repeatedly proposed as an antivirulence strategy that promotes *S. aureus* dispersal (Li et al., 2011; Khan et al., 2015; Piewngam et al., 2018). Although the present study does not identify the LAB-derived molecules responsible for this effect, the repression of *agr*-associated targets is consistent with the broader concept that probiotic or commensal bacteria can attenuate staphylococcal virulence by interfering with quorum-sensing-dependent outputs (Bui et al., 2025; Gless et al., 2025; Wang et al., 2025). Nevertheless, this response should be interpreted with caution, because the *agr* system has a complex role in biofilm biology: while it controls multiple virulence factors, it can also contribute to biofilm dispersal depending on the physiological state and environmental context (Boles and Horswill, 2011; Bronesky et al., 2016).

The repression of *icaA* suggests reduced investment in matrix production, since the *icaADBC* operon mediates the synthesis of one of the major polysaccharide components of the staphylococcal biofilm matrix, PNAG/PIA (François et al., 2023; Peng et al., 2023). Similarly, the downregulation of *sfaA*, which encodes the exporter required for staphyloferrin A secretion, may indicate altered siderophore deployment or reduced iron-scavenging capacity in the competitive mixed biofilm environment (Hannauer et al., 2015; Nakaminami et al., 2017). These changes are relevant in the context of mastitis, as biofilm formation and iron acquisition are key traits contributing to *S. aureus* persistence in host-associated niches, and our previous characterization of human milk-associated *S. aureus* showed that mastitis-derived isolates, including SA4 and SA55, display strong biofilm-forming capacity and siderophore-associated traits (Jurado et al., 2025a). The repeated induction of *uspA* across LAB treatments suggests that *S. aureus* activated a general stress-adaptation response, potentially favoring survival under adverse conditions such as nutrient limitation, acidic or metabolic stress, oxidative stress, or local physicochemical changes generated during interspecies competition (Even et al., 2009; Mujchariyakul et al., 2026). Taken together, these data support the idea that LAB-mediated displacement of *S. aureus* biofilm cells is not merely associated with an immediate loss of viability, but with a progressive transcriptional transition toward reduced expression of virulence-, matrix- and iron-acquisition-related functions, while stress-adaptation mechanisms are activated as the pathogen loses dominance within the mixed biofilm community.

Subsequently, RNA-seq was used to determine whether these targeted changes reflected a broader transcriptional remodeling. The RNA-seq analysis of SA4 biofilms exposed to LAB for 16 h confirmed that the repression of matrix- and iron-related functions and the induction of stress-adaptation markers were part of a much broader transcriptional response of SA4 under the tested conditions. A large *core* of transcriptional signatures was observed across the three LAB treatments, indicating that exposure to the different probiotic candidates elicited a convergent transcriptional response in SA4 biofilm cells. This shared response was characterized by activation of glycolytic, respiratory, arginine-catabolic, oxidative-stress and proteostasis-associated functions, together with repression of genes involved in canonical biofilm formation, surface-associated traits, cell-envelope maintenance, metal acquisition and host-interaction functions. Therefore, under the tested conditions, LAB exposure appeared to impose broad ecological pressure on SA4 biofilm cells, promoting a coordinated repression of classical biofilm-, surface- and virulence-associated functions while driving this strain toward a stress-adapted survival physiology. This interpretation is consistent with previous works showing that environmental and microbial pressures can reshape *S. aureus* metabolism, stress responses and virulence regulation in a coordinated manner, rather than affecting these processes independently (Even et al., 2009; Cretenet et al., 2011; Mujchariyakul et al., 2026).

Importantly, this conserved *core* did not exclude the existence of LAB strain-dependent transcriptional signatures. This is relevant because probiotic antagonism against *S. aureus* is generally considered strain-dependent, reflecting differences in secreted metabolites, bacteriocin-like compounds, surface structures, stress tolerance, nutrient competition and quorum-sensing interference capacity (Kang et al., 2017; Bui et al., 2025). In this study, *L. fermentum* I7 was associated with additional repression of adhesion-, competence- and scavenging-related traits, *L. reuteri* 7SNG3–30 with a more focused response involving membrane/envelope homeostasis and metabolic adjustment, and *L. salivarius* 22SNG3–30 with a more regulatory and stress-adaptive signature together with repression of selected biofilm- and surface-associated functions. This strain-dependent behavior is in agreement with previous studies showing that LAB-pathogen interactions in mixed biofilms are highly dependent on the tested strain (Jara et al., 2022; Jurado et al., 2025b).

This global transcriptional response of SA4 should also be interpreted considering the intrinsic heterogeneity of *S. aureus* biofilms. Biofilm communities contain cells in different physiological states, and recent single-cell RNA-seq studies have shown that *S. aureus* biofilms include transcriptionally diverse subpopulations with distinct metabolic, stress-response, virulence and persistence-associated profiles (Korshoj and Kielian, 2024; Yan et al., 2024). Therefore, the bulk RNA-seq profile obtained here should be understood as an aggregate population-level response after 16 h of LAB exposure, which may mask the coexistence of subpopulations with different degrees of metabolic activity, stress adaptation or susceptibility to LAB pressure. This supports the interpretation of the 16-h sampling point as a transitional phase during biofilm displacement, rather than as a uniform physiological state shared by all SA4 cells within the biofilm.

RT-qPCR and RNA-seq approaches were in agreement for many *S. aureus* targets and, particularly, for genes displaying larger transcriptional changes, such as *icaA*, *sfaA* and *uspA*. This agreement is relevant because RT-qPCR and RNA-seq differ in normalization strategy, dynamic range, primer or gene-model dependency and statistical framework. Previous benchmarking studies comparing RNA-seq workflows with RT-qPCR data showed that, although global correlations are usually high, a proportion of genes may display non-concordant differential-expression trends depending on the method used (Everaert et al., 2017; Coenye, 2021).

The transcriptomic profiles of the LAB strains provided complementary information about how the probiotic candidates behaved during interaction with SA4 biofilms. Overall, the LAB responses were more moderate and strain-dependent than the transcriptional responses observed in SA4 biofilm cells. However, this should not be interpreted as evidence of limited biological activity. Differential expression was calculated relative to the corresponding LAB single-species biofilm controls; therefore, functions that were already active during LAB biofilm growth, including basal fermentative metabolism, organic acid production, nutrient acquisition or other antagonistic traits, would not necessarily appear as interaction-specific transcriptional changes. In fact, the LAB transcriptomic responses involved pathways related to carbohydrate metabolism, amino-acid and nutrient acquisition, nucleotide metabolism, translation, surface/envelope adaptation and redox- or stress-associated functions, supporting the idea that these strains adjusted their physiology to persist and overgrow within the mixed biofilm environment (Papadimitriou et al., 2016).

Within this general pattern, *L. fermentum* I7 and *L. reuteri* 7SNG3–30 showed a broader interaction-associated transcriptional remodeling than *L. salivarius* 22SNG3-30. In *L. fermentum* I7, the induction of genes related to translation, biosynthetic activity, nutrient transport and surface/envelope adaptation, together with repression of selected stress/detoxification, arginine biosynthesis/regulatory, and mobile-element-associated functions, is compatible with an actively maintained population rather than with a strong acute stress response. *L. reuteri* 7SNG3–30 displayed the broadest LAB transcriptional response, involving nucleotide biosynthesis, nutrient transport, translation-associated functions, surface/envelope adaptation and redox or pH-homeostasis adjustments. This profile suggests a more dynamic adaptive response to *S. aureus* presence, consistent with an ecological competition model in which *L. reuteri* 7SNG3–30 colonizes the preformed staphylococcal biofilm while adjusting nutrient acquisition, biosynthesis and envelope-related functions. This interpretation is also supported by transcriptomic studies showing that *L. reuteri* can remodel purine/nucleotide metabolism, ABC transporters, amino-acid transport, arginine-related metabolism, membrane-associated functions and stress-response pathways in response to nutritional or environmental challenges (Zhu et al., 2022; Josephs-Spaulding et al., 2024; Liu et al., 2024). Therefore, although neither *L. fermentum* I7 nor *L. reuteri* 7SNG3–30 showed a response dominated by a single classical antimicrobial pathway, their transcriptomic profiles indicate active physiological adaptation during coexistence with SA4 biofilm cells under the tested conditions.

In contrast, *L. salivarius* 22SNG3–30 showed a much more restricted differential transcriptional response, characterized by limited changes in carbohydrate metabolism and surface/envelope-associated functions, together with repression of stress-, regulatory- and mobile/secreted-function-associated genes. These functional categories are consistent with previous studies showing that *L. salivarius* can remodel carbohydrate metabolism, osmoregulation, cell-surface properties and envelope-associated functions in response to environmental challenges (Wang et al., 2020; Kobierecka et al., 2023). One possible interpretation of the comparatively low number of differentially expressed genes is that, by the 16-h RNA-seq sampling point, *L. salivarius* 22SNG3–30 had already established itself efficiently within the biofilm. This interpretation is supported by the kinetic data from the present study, where *L. salivarius* 22SNG3–30 maintained higher biofilm-associated counts than the other LAB strains throughout the experiment. In parallel, this condition was associated with a greater reduction and consistently lower biofilm-associated *S. aureus* SA4 counts throughout the treatment period, suggesting that the main adaptive or competitive response of *L. salivarius* 22SNG3–30 may have occurred before the 16-h RNA-seq sampling point. Therefore, the low number of differentially expressed genes in *L. salivarius* 22SNG3–30 does not necessarily indicate low activity but a state of previous dominance. Instead, it may reflect a transcriptional state closer to homeostasis within the single-species biofilm, rather than an acute, ongoing response to interspecies interaction at the sampled time point.

These population dynamics should also be interpreted within the context of the experimental model. Clinical studies have reported considerable variability in the bacterial loads and microbial composition of milk from women with mastitis (Arroyo et al., 2010; Jiménez et al., 2015), and measurements obtained from milk samples may not necessarily reflect the heterogeneous bacterial populations attached to the mammary duct epithelium. Moreover, the concentrations reached by orally administered probiotic strains within the mammary environment have not been consistently established (Arroyo et al., 2010). Consequently, a physiologically representative or therapeutic LAB-to-*S. aureus* ratio cannot be accurately defined. The initial ratios used here (approximately 1:2,400 for SA4 and 1:3,700 for SA55) were therefore selected to establish a stringent competition model in which a comparatively low LAB inoculum was challenged against a mature and densely populated *S. aureus* biofilm, rather than to reproduce an anticipated therapeutic dose. Environmental acidification should also be considered when interpreting the observed response. In our previous study, 24-h cell-free supernatants from these same LAB strains reached pH values of 3.76–4.17, compared with an initial MRS pH value of 6.14, and showed antibiofilm activity against SA4 and SA55 (Jurado et al., 2025b). These findings indicate that the accumulation of lactic acid and other organic acids and the resulting environmental acidification may have contributed to the antibiofilm activity observed here. As the present study was designed to characterize the global effect of active LAB cultures on *S. aureus* biofilms, the specific relative contributions of acidification and other LAB-derived metabolites warrant further investigation.

Beyond these mechanistic aspects, several limitations should be acknowledged. This study was performed in a static *in vitro* biofilm model, which allowed controlled analysis of the interactions between active LAB cultures and preformed *S. aureus* biofilms but does not reproduce the full complexity of the lactating mammary gland, including milk flow, immune factors, epithelial surfaces and host-derived nutritional constraints. In addition, RNA-seq was performed only with SA4 at the 16-h time point, which was selected as a biologically informative stage of transcriptional transition but does not capture the entire temporal sequence of biofilm displacement or the possible strain-specific response of SA55. Another limitation is that read mapping was performed using available reference genomes rather than strain-specific genomes for the isolates used here, which may have affected gene assignment and differential-expression interpretation. Finally, transcriptomic changes alone do not demonstrate metabolite production or functional causality. Future work should therefore combine time-resolved RNA-seq, metabolomics, pH-controlled experiments and purified metabolites to distinguish the contributions of environmental acidification and other LAB-derived factors to *S. aureus* biofilm persistence. In parallel, the most promising strains should be further evaluated in more physiologically relevant *in vitro* models, including human mammary epithelial cell cultures with secretory features, to more closely mimic the host environment. These approaches would help prioritize candidate strains for subsequent validation in appropriate animal models and, ultimately, in well-controlled clinical studies in lactating women.

## 5 Conclusions

This study shows that the three LAB strains tested were able to colonize and progressively displace preformed *S. aureus* biofilms formed by the two mastitis-associated strains, SA4 and SA55. This antibiofilm effect was not immediate but followed a time-dependent competitive transition in which LAB became established within the biofilm before the attached staphylococcal population declined. Targeted RT-qPCR analysis of both SA4 and SA55 showed a time-dependent transcriptional response that was generally more pronounced after 16 h of LAB exposure and in SA4. RNA-seq analysis, performed with SA4 after 16 h of exposure, revealed extensive transcriptional remodeling, including repression of genes and pathways associated with quorum sensing, virulence, matrix production, surface-associated traits and metal acquisition, together with activation of stress-, energy- and proteostasis-related functions. Exposure to the three LAB strains elicited a shared transcriptional response in SA4, while each LAB-SA4 interaction also displayed pair-specific transcriptional signatures. In contrast, the transcriptomic responses of the LAB strains were more moderate and strain-dependent. Among the strains tested, *L. salivarius* 22SNG3–30 showed a particularly promising phenotypic profile, characterized by rapid biofilm colonization, high attached-cell counts and an earlier reduction of the *S. aureus* SA4 population. However, these *in vitro* findings do not demonstrate clinical efficacy, and the potential of the tested LAB strains for mastitis-associated applications requires further validation in host-cell and animal models, followed by well-controlled clinical studies. Overall, these findings support further investigation of LAB as potential complementary tools to weaken *S. aureus* biofilm persistence in the context of acute lactational mastitis.

## Data Availability

The datasets presented in this study can be found in online repositories. The names of the repository/repositories and accession number(s) can be found below: https://www.ncbi.nlm.nih.gov/geo/, GSE337562.
